# Exploring the Antioxidant Potential of Gellan and Guar Gums in Wound Healing

**DOI:** 10.3390/pharmaceutics15082152

**Published:** 2023-08-17

**Authors:** Gianina Dodi, Rosina E. Sabau, Bianca E.-B. Crețu, Ioannis Gardikiotis

**Affiliations:** 1 Biomedical Sciences Department, Faculty of Medical Bioengineering, Grigore T. Popa University of Medicine and Pharmacy of Iasi, 9-13 Kogalniceanu Street, 700454 Iasi, Romania; rosina.sabau@gmail.com; 2 Department of Natural Polymers, Bioactive and Biocompatible Materials, Petru Poni Institute of Macromolecular Chemistry, 41 A Grigore Ghica Voda Alley, 700487 Iasi, Romania; cbiancaelenabeatrice@yahoo.com; 3 Advanced Research and Development Center for Experimental Medicine, Grigore T. Popa University of Medicine and Pharmacy of Iasi, 9-13 Kogalniceanu Street, 700454 Iasi, Romania; ioannis.gardikiotis@umfiasi.ro

**Keywords:** antioxidant activity, gellan gum, guar gum, DPPH assay, wound healing

## Abstract

It is acknowledged that the presence of antioxidants boosts the wound-healing process. Many biopolymers have been explored over the years for their antioxidant potential in wound healing, but limited research has been performed on gum structures and their derivatives. This review aims to evaluate whether the antioxidant properties of gellan and guar gums and wound healing co-exist. PubMed was the primary platform used to explore published reports on the antioxidant wound-healing interconnection, wound dressings based on gellan and guar gum, as well as the latest review papers on guar gum. The literature search disclosed that some wound-healing supports based on gellan gum hold considerable antioxidant properties, as evident from the results obtained using different antioxidant assays. It has emerged that the antioxidant properties of guar gum are overlooked in the wound-healing field, in most cases, even if this feature improves the healing outcome. This review paper is the first that examines guar gum vehicles throughout the wound-healing process. Further research is needed to design and evaluate customized wound dressings that can scavenge excess reactive oxygen species, especially in clinical practice.

## 1. Introduction

The wound-healing dynamic process, measurement of antioxidant properties in plant-derived complexes, and customized wound dressing design have historically been the focus of interest for many scientists, mainly in separate stages, but limited as a flow. It is well known that any error in the epithelization process results in abnormal wound healing that generates cascading side effects, such as physical and mental health degeneration of the patient, financial burdens for health systems, etc. [1]. Therefore, when thinking about the complexities of ideal treatment in all types of wounds, researchers have focused lately on exploring the potential of nature’s intelligence to create biomimetic ingredients [2]. For this reason, in the last decades, the desired properties of a good wound-healing agent follow the biomaterial intrinsic features that induce keratinocyte proliferation and differentiation [3]; stimulate fibroblast proliferation and collagen formation; and exhibit antimicrobial, antioxidant and anti-inflammatory benefits [4]. In most cases, the biomimetic dressing should exhibit at least two or more of the above assets. 

Antioxidant activity, the most commonly used term for the capacity to inhibit all molecules with high redox potential, has been intensively studied in the last decade since it tackles the role of oxidative stress in numerous diseases [5]. In 2010, Hajhashemi et al. [6] evaluated the position of antioxidants in disease prevention based on controversial basic research and clinical studies that suggest their potential to prevent oxidative damage. The collected data recognises the free radicals and radical-derived reactive oxygen species (ROS) in moderate concentration as regulatory mediators in signalling processes, but vitamins E, C, and beta-carotene do not have the expected effect. Only selenium is suggested to reduce cancer incidence, but further research is needed for a valid outcome [7]. In 2020, Sharifi-Rad et al. [8] introduced a balanced lifestyle and nutrition as controlling factors of oxidative stress in various diseases. The authors highlight the need for large randomised trials on the benefits or risks of antioxidant plant-derived bioactive molecules during treatment for cardiovascular disease, cancer, diabetes, and neurodegenerative disorders. More recently, Rudrapal et al. [9] focused on phenolic acids, flavonoids, tannins, catechins, lignans, anthocyanidins, and stilbenes and their role in oxidative stress-induced human diseases. The antioxidant capacity of the polyphenols reported in plants and foods fights against oxidative stress-induced disorders, mostly on the in vitro level for the prevention of cardiovascular and neurodegenerative diseases, diabetes, cancer, inflammation-related diseases, and infections. However, further pre-clinical and clinical investigations are still needed after years of search. Overall, it is essential that antioxidant activity is studied in the future to better understand and manage various diseases. 

Bearing in mind these experiences, this work proposes the evaluation of antioxidant activity in natural compounds, namely gellan and guar gum related to their wound-healing potential. It is important to mention that this is the first review paper that evaluates guar gum as a wound dressing.

## 2. Methods

**Figure 1:**
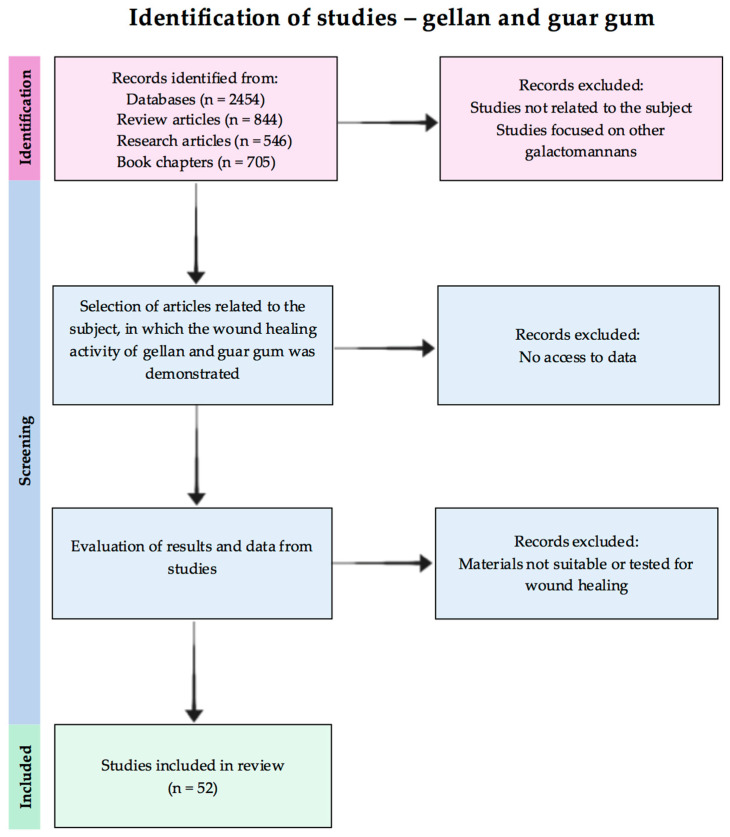
Flow chart of the reviewed studies selection.

A literature search was conducted in several databases (PubMed.gov, Science Direct, Web of Science, ResearchGate, Scopus) using the following keywords: [gellan/guar gum and wound healing], [gellan/guar and wound healing]; [gellan/guar gum and antioxidant activity and wound healing], [gellan/guar and antioxidant activity], [galactomannan, gellan/guar gum, wound healing]. The selection criteria for the included studies are the following: issued in English, relevant studies and data regarding wound healing activity of the guar and gellan gums (see Figure 1). For gellan gum, the authors included the relevant papers based on the above-mentioned keywords from the period 2014–2023, and guar gum for all times.

After the manual examination of the articles, those suitable for the subject of the review were selected. Additionally, the reference lists of the included papers were carefully searched for any additional noteworthy material.

## 3. Antioxidant Activity Future in Wound Healing

Wound healing is a dynamic phenomenon involving a synchronized sequence of well-orchestrated biochemical and cellular activities to achieve tissue regeneration [10]. This process of growth and regeneration of damaged tissue can be divided into four differentiated, though overlapping stages: hemostasis, inflammation, proliferation (neo-angiogenesis, granulation, re-epithelialization), and extracellular matrix remodelling [11]. In brief, the blood coagulation phase points to the formation of platelets and fibrin, shadowed by the inflammatory response due to the recruitment of neutrophils and monocytes, the release of growth factors and cytokines, and lymphocyte infiltration that provides protection against bacteria. Overlapped with the inflammatory phase, the proliferation boosts the re-epithelialization, the deposition of the extracellular matrix, new blood vessels, collagen, and fibroblasts. The establishment of tissue granulation and the tissue-remodelling phase restores the epidermal barrier by keratinocytes migration, proliferation, and differentiation [2]. However, in all these chronologically overlapping phases, an essential component of wound nutrition is oxygen (O_2_), vital for tissue renewal and wound infection reduction, as described by Thomas Hunt, a pioneer in the field of oxygen and wound healing [12]. A new paradigm was described in his search for the mechanisms by which O_2_ and wound healing are linked. Namely, the wound area cells are tailored with a specialized enzyme that converts O_2_ to ROS [13].

Radical derivatives of O_2_, acknowledged as ROS with their family, both free radical and non-free radical molecules, such as superoxide anion-O_2_^−^, peroxide-O_2_^−2^, hydrogen peroxide H_2_O_2_, hydroxyl radicals -OH, and hydroxyl OH^−^ ions, perform as messengers in the wound-healing response, since a number of cells utilize these radicals, starting from hemostasis and continuing up to tissue repair [14]. 

As described by Dunnil et al. [15], the functional role of ROS is briefly mentioned here: early ROS mediates hemostasis tailing platelet exposure to the extracellular matrix (ECM), signals rapid migration of neutrophils towards wound site, stunts bacterial growth through H_2_O_2_ release, further signals the monocytes infiltration, and finally promotes endothelial cell division, angiogenesis, and the formation of fibroblasts and collagen.

The level of ROS involved in the repair process mediates the response from normal cell functioning at the basal level, continuing up to cell death and cellular necrosis in excessive ROS induction, leading to impaired healing [16]. The increased ROS level is mainly attributed to the reactive nitrogen, iron, copper, and sulfur species that induce oxidative stress and impair the redox balance [17]. In the context of an imbalance between the production and destruction of ROS (excessive level), defined as oxidative stress, the antioxidant-specific proteins kick in and donate their own electrons, consequently avoiding capturing electrons from DNA, proteins, and lipids. In 2008, Rodriguez et al. [18] validated the hypothesis that the final outcome in wound healing depends on the balance between low stimulating effects and high levels of ROS, which are translated into cellular damage and impaired wound repair. Therefore, an effective wound-healing strategy also involves antioxidant activity management. 

However, how is the antioxidant activity determined? Many attempts were evaluated over the years to generate a universal measure of antioxidant activity, and currently, the best choice of antioxidant assay is a controversial challenge. 

The review of Munteanu and Apetrei [19] from 2021 and the review paper of Christodoulou et al. in 2022 [20] present the most important tests used to determine the antioxidant activity, along with the detection mechanism, applicability, advantages, and disadvantages. The most used procedures to quantify the antioxidant activity were classified into distinct categories, namely, spectrometry (UV-VIS, fluorescence, chemiluminescence), electrochemical assays (voltammetry, amperometry, and biamperometry), liquid and gas chromatography, antibody, and electrophoretic techniques [21,22]. Spectrophotometric methods characterized by sensitivity, reproducibility, rapidity, reliability, simplicity, and low cost [20], determine the total antioxidant activity (TAC) and total phenolic content (TPC) as follows:

- A [2,2-di(4-tert-octylphenyl)-1-picrylhydrazyl] (DPPH) test measures the antioxidant reaction with free organic radicals, founded on the neutralisation of the DPPH radical due to donated electrons from the antioxidants, with colour change at 517 nm from purple to yellow; the DPPH radical scavenging activity is often expressed using the following equation [23]:
  (1) $$DPPH\;radical\;scavenging\;activity\left(\%\right)=\frac{A_{control}-A_{sample}}{A_{control}}\times 100$$
  where A_control_ and A_sample_ are absorbance values of a control mixture without antioxidant and a mixture containing antioxidant, respectively.
  The EC_50_ (the efficient concentration of the antioxidant necessary to reduce the initial DPPH concentration by 50%) and TEC_50_ (the necessary time to reach the equilibrium state with EC_50_) values are also used to measure the antioxidant activity of the DPPH scavenging method.
- A Folin–Ciocalteu assay is based on the reductive capacity of antioxidants to determine the TPC in an alkaline state; the Folin–Ciocalteu reagent contains a mixture of phosphomolybdate and phosphotungstate that are reduced to generate a blue colour chromophore when it reacts with phenols with the maximum absorption at 765 nm. This single electron transfer reaction-based method uses gallic acid, catechin, caffeic acid, chlorogenic acid, or ferulic acid as reference standards, and the TPC values are usually given, for example, as gallic acid equivalents (GAE) [24].
- A cupric reducing antioxidant power (CUPRAC) test measures the TAC of the reduction of Cu (II) light blue to Cu (I) yellow-orange by antioxidants at 450 nm; the CUPRAC activity is expressed either as % inhibition or the equivalent of Trolox, gallic acid, ascorbic acid, quercetin, or α-tocopherol, used as standard compounds [25].
- A ferric reducing antioxidant power (FRAP) test measures the antioxidant potential through the reduction of the colourless complex of ferric ions (Fe (III)) to the intensely blue ferrous complex (Fe (II)) with tripyridyltriazine or potassium ferricyanide by antioxidants at 593 nm in acid environments; the activity is expressed as micromolar equivalents of ferrous ions or in relation to a standard antioxidant [26].
- A 2,2′-azinobis-(3-ethylbenzothiazoline-6-sulfonic acid) (ABTS) test measures the relative ability of antioxidants to neutralise the blue-green ABTS stable radical cation generated in the aqueous phase, and quantified at 734 nm; the antioxidant activity is dependent on the degree of discolouration of the blue-green colour [22].
- Thiobarbituric acid reactive substance (TBARS) procedures are centered on the reactivity of malondialdehyde (MDA) with TBA to produce a red colour quantified at 535 nm; the technique first complexes ethylenediaminetetraacetic acid (EDTA) with Fe (II), then interacts with hydrogen peroxide to produce HO• radical, which reacts in the presence of ascorbic acid with the deoxyribose sugar to produce by-products. At low pH values, the mixture forms MDA in combination with TBA, followed by MDA-TBA chromogen. The scavenging activity is measured based on the inhibition of deoxyribose degradation [27].
- A ferrous oxidation xylenol (FOX) test measures the levels of hydrogen peroxide in biological systems by the oxidation of Fe (II) to Fe (III), which is then reacted with xylenol orange (XO) to generate a ferric-XO complex, quantified at 560 nm with a blue-purple colour [28].
- A ferric thiocyanate (FTC) assay also measures the levels of hydrogen peroxide as the ferric ion is converted by an oxidant from a ferrous ion, similar to FOX, but in this case, the ferrous ions are monitored by the thiocyanate complex, quantified at 500 nm with a blood-red colour; the test identifies and quantifies the total phenolic content, lipid oxidation, and flavonoids using mainly gallic acid as a standard [29].
- A β-carotene bleaching assay quantifies pro-oxidants and antioxidant species by measuring the levels of peroxyl radicals that combine with β-carotene to form a stable carotene radical, thus reducing the concentration of β-carotene in a testing solution; if an antioxidant is present in the solution, β-carotene competes for the formation of the carotene radical, followed by the decolourization of the orange-yellow or dark-yellow carotene solution verified at 440 nm [30].
- Superoxide radical scavenging and nitric oxide radical scavenging assays determine both the total oxidant scavenging capacity of antioxidants. First, one measures the bright blue colour of formazan at 560–562 nm produced by nitroblue tetrazolium (NBT) light-yellow salt after it is reduced by oxygen as the tetrazole ring is broken, triggering dismutation. In the second test, nitric oxide radicals are determined by aqueous sodium nitroprusside (SNP) solution, react with oxygen to form nitrite ions, and with sulfanilic acid for diazonium ion, then are linked with N-(1-naphthyl) ethylenediamine (NED) to produce a red azo dye [31].
- The oxygen radical absorption capacity (ORAC) and the hydroxyl radical antioxidant capacity (HORAC) assays rely on the antioxidant reaction with peroxyl radicals induced by 2,2′-azobis-2-amidino-propane (AAPH) and quench OH radicals generated by a Co (II)-based Fenton-like system [32].
- Hydrogen peroxide scavenging and peroxynitrite scavenging activity assays establish the total oxidant scavenging capacity of antioxidants in fluorescence, using horseradish peroxidase (HRP) enzyme and scopoletin or dihydrorhodamine to measure the fluorescence intensity or inhibition [33].
- The total peroxyl radical trapping antioxidant parameter (TRAP) calculates the antioxidant capacity to scavenge luminol-derived radicals, generated from AAPH decomposition in chemiluminescence [34].
- A catalase assay (CAT) demonstrates the presence of catalase, an enzyme that dissociates hydrogen peroxide into molecular oxygen and water; the catalase activity is calculated based on the rate of decomposition of hydrogen peroxide, proportional to the reduction in the absorbance at 240 nm [35].
- Glutathione peroxidase (GSH-Px) catalyses the reduction of hydrogen peroxides with the aid of GSH to produce alcohol, oxidized glutathione, and water; the absorbance level measured by UV-VIS at 340 nm is directly proportional to the GPx activity in the sample [36].
- Superoxide dismutase (SOD) converts the superoxide anion to H_2_O_2_, which is a substrate for CAT and GSH-Px; the SOD activity is determined as the inhibition or reduction of water-soluble formazan dye at 450 nm, for the detection of superoxide radicals generated by xanthine oxidase and hypoxanthine [37].
- A GSH content assay involves oxidation of GSH by the sulfhydryl reagent 5,5′-dithio-bis (2-nitrobenzoic acid) to form the yellow derivative 5′-thio-2-nitrobenzoic acid, measurable at 412 nm [38].

As observed, these chemical and enzymatic tests used to determine the antioxidant capacity are indeed handy, relatively rapid, reproducible, and partially automated, developed primarily to screen and assess new antioxidant compounds, materials, or the extracts’ products and by-products, but the selection of appropriate assays depends on the type of tested antioxidants, either hydrophilic or hydrophobic, solvent solubility, working pH, temperature, absorbance interferences triggers, specificity of the substrate, and instability of the compound. In this context, there is an imperative need to select the applicable assay or a combination of assays in each case for each type of antioxidant potential support. 

In the following section, this paper focuses on the wound-healing potential of two types of gums, one intensively used in the field, namely, gellan gum, and guar gum, which is a novice in it, along with the antioxidant activity located in the preliminary phase. 

## 4. Gums

Biopolymers such as cellulose, chitosan, collagen, gelatin, and gums (gellan gum, xanthan gum, dextran, etc.) were considered to be a matter of interest in regenerative medicine in the past years, being the essential component materials used in the treatment of wounds [2]. Currently, these natural polymers are still being explored worldwide due to their interesting properties which include non-toxicity, biocompatibility, simulation of the original extracellular matrix (ECM), non-immunogenicity, natural abundance, low cost processing, and the presence of desirable functionalities [39].

Natural gums, the most abundant industrial raw materials, are polysaccharides comprising glucose, galactose, mannose, arabinose, rhamnose, xylose, and uronic acid sugar units produced by plants. Similar to biopolymer features, gums are preferred over comparable synthetic materials, mainly due to their multi-functionality, non-toxicity, biocompatibility, biodegradability, hydrophilicity, surface flexibility, low cost, and availability [40]. 

Gums are obtained from various sources, such as microorganisms by fermentation, marine algae by in vitro enzymatic process, and plants and animals by extraction in large amounts [41]. The major gums synthesized from microbes are glycan, pullulan, dextran, xanthan, and gellan. Those with marine origin are alginate, agar, and carrageenan. Those from animals (chitin, chitosan, hyaluronic acid, and chondroitin sulphate) and from plants are seed gums (locust bean, guar, cellulose and amylase), tree exudates (gum arabica, tragacanth, gum ghatti, karaya), tubers (starch), and extracts (pectin, larch gum, roots) [42,43].

**Figure 2:**
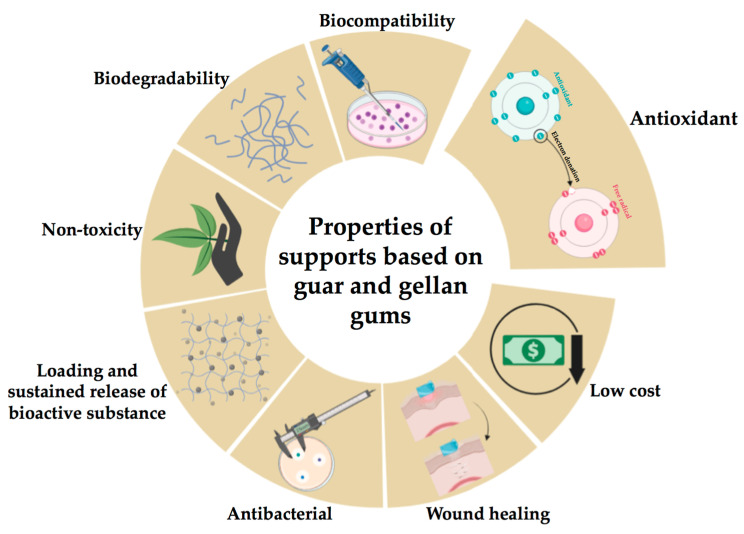
Key properties of the gellan and guar gums (created with BioRender.com (accessed on 1 August 2023)).

Figure 2 presents the key properties of gellan and guar gums surveyed in the following sections.

### 4.1. Gellan Gum and Its Derivatives 

Gellan gum is a linear, negatively charged, high molecular weight exopolysaccharide, consisting of four saccharide-repeating units of two β-D-glucose, one β-D-glucuronate, and one α-L-rhamnose, meaning 60% of glucose, 20% of rhamnose, and 20% of glucoronate [44]. It is obtained by bacterial exopolysaccharide fermentation from *Pseudomonas elodea*, *Sphingomonas elodea* or *Sphingomonas paucimobilis*, followed by neutratisation using KOH or NaOH, filtration to obtain deacetylated gellan, followed by precipitation with isopropanol, washing with acetone and ether, dissolution in water, dialysis, and finally, drying at 55° or lyophilization. 

The main property of gellan gum is the ability to produce steady and translucent gels, stability in acidic pH, property dependent on the pH, temperature, type of cations in solution, and polymer concentration. At low temperatures, gellan molecules arrange in well-organized double helices, and when heated the polymer resembles a roughly coiled shape, a property known as thermally reversible gelation. Other characteristics include malleability and versatile texture that allow adjustable gel elasticity, biocompatibility, biodegradability, non-toxicity, high efficiency, mucoadhesive features, and outstanding thermal and acid stability [45]. Subsequently, this polysaccharide has attracted great attention in a variety of fields, including biotechnology, cosmetics, food, and of course from a biomedical standpoint. In wound dressing design, gellan gum uses most of the above parameters suitable to promote the proliferation of cell growth, biocompatibility with human skin fibroblast cells, and resistance to enzymatic action [46]. 

As already mentioned, gellan gum has been intensively employed in the wound dressing scaffold design in different forms [47]; therefore, numerous publications are available in specialised databases. For example, in the electronic platform PubMed, the search for gellan gum that has been scientifically demonstrated to have wound healing activity returned 45 publications, from which 6 reviews were published from 2018 and 2023, with only 1 specific to wounds, to the best of our knowledge. The review paper of Feketshane et al. [46] focuses on the unique physicochemical and biological features of wound dressing scaffolds prepared from gellan gum. The authors point out the advantages of gellan gum-based wound dressings and the encountered limitations in the available studies. Among these restraints, it is important to mention the need for more preclinical research studies, the design of various formulas of wound dressings based on gellan gum, such as bandages, patches, foams, loaded or not with therapeutic agents in order to reach clinical application for wound treatment [46].

Table 1 describes gellan gum-based wound dressings developed in the period 2014–2023 that have shown promising results, based on the formulation type and obtaining method, encapsulated ingredients, performed assays, and the antioxidant capacity related to the rate of wound closure. 

**Figure 3:**
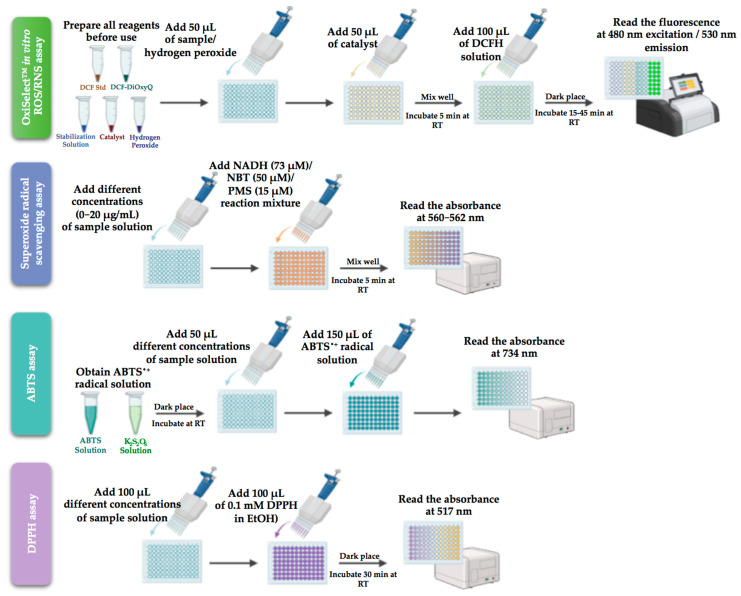
Key antioxidant assay used for gellan and guar gums systems (created with BioRender.com (accessed on 1 August 2023)).

Apigenin-loaded gellan gum–chitosan hydrogels designed by Shukla et al. [50] by covalent cross-linking were evaluated for their antioxidant potential in granuloma tissues, collected from a full thickness wound. The antioxidant level was calculated using catalase, SOD assay, and GSH level by measuring the supernatant absorbance at 412 nm using a UV spectrophotometer (see protocols in Figure 3). According to the obtained results, the hydrogels exhibit antioxidant activity in the granuloma tissues during the wound-healing process on the ninth day in diabetic wounds. The significant improvement in the SOD, GSH, and CAT levels protects the cell membrane against oxidative damage by controlling free radical production. 

The antioxidant features and the radical scavenger potential of Eumelanin incorporated into gellan gum spongy-like hydrogels prepared by ionic cross-linking were confirmed by the reduced amount of ROS/RNS, as determined by OxiSelect™ in vitro ROS/RNS assay [49] (protocol in Figure 3). This commercially available kit measures the total free radical presence of a sample based on the reaction of ROS and RNS species with 2′, 7′-dichlorodihydrofluorescein diacetate (DCFH). This was the first study on gellan gum-melanin spongy-like hydrogels applied in a skin wound healing model.

DPPH scavenging assay, as described in Figure 3, analysed the antioxidant activity of carboxymethyl cellulose nanofibril-loaded fucoidan/alginate-based gellan gum hydrogel and co-encapsulated ofloxacin, tea tree or lavender oil in gellan gum-based hydrogel films both tested for the treatment of full-thickness wounds. The fucoidan/alginates/carboxymethyl cellulose hydrogel promotes healing since it induces a higher rate of apoptosis by stimulating ROS generation. The treatment with laser-irradiated hydrogel reduced inflammation, induced cell proliferation and differentiation, and increased apoptosis, boosting the wound-healing process [52].

The transparent and flexible gellan gum hydrogel films loaded with essential oil and prepared by the solvent casting ionotropic gelation method displayed slightly similar antioxidant activity, as determined by DPPH assay with pure oils, meaning that oils retain their antioxidant activity after incorporation into the polymeric matrix. The findings in this paper demonstrate that hydrogel films promote cellular growth and proliferation in wound treatment [56].

Recently, the single-type antioxidant assay was updated with a combination of tests, in the name of DPPH and ABTS radical scavenging assay that showed a similar trend, dose-dependent antioxidant capacity, in fact, as the concentration increases, the ability to eliminate radicals increases when testing dopamine and PEG functionalized gellan gum injectable hydrogel [60]. 

Figure 3 displays the used protocols for antioxidant activity determination for gellan and guar gums according to Table 1 and Table 2.

### 4.2. Guar Gum and Its Derivatives

In recent years, the interest in guar gum hydrophilic polysaccharide has gained considerable attention in many fields, as a binder, disintegrant, suspending, thickening, and stabilizing agent. Guar gum is derived from guar bean seeds of the plant *Cyamopsis tetragonoloba,* cultivated mainly in India and Pakistan and also in the Southern Hemisphere in semi-arid zones in Brazil, South Africa, Australia, Texas, and Arizona. It is known as galactomannan, since it contains nearly 80% galactomannan, along with 12% water, 5% protein, 2% insoluble ash, 0.7% ash, and 0.7% fat, and appears as a white-to-yellowish white odourless powder with a bland taste.

The gum is extracted from guar seeds by different processing techniques, as follows: First, the seeds are split into germs by separation from the endosperm, followed by a thermal dehusking process to obtain the guar splits [70]. Then, the refined guar splits obtained by milling are either packed as guar gum products or are further treated by dissolution, filtration to remove the insoluble parts, precipitation with alcohol, another filtration step, and are then dried and passed through a mill with controlled size in order to achieve various grades of guar gum [70].

Guar gum is basically composed of a straight chain of D-mannose units, united by β (1–4) glycoside linkages, bearing a single D-galactose unit on approximately every alternate mannose joined to it by an α (1–6) glycoside linkage [71]. Guar gum consists of non-ionic polydisperse rod-shaped molecules made up of about 10,000 residues. The ratio of mannose to galactose units is approximately 1.5:1 or 2:1, with the distribution related to the solubility behaviour of guar gum [28]. It is worth mentioning that guar gum is partially soluble in water due to protein residues, but hydrates quickly to form a viscous solution. The high galactose substitution intensifies the solubility, dispersiveness, emulsification, and stiffness, but lowers the extensibility of the isolated chains since the galactose residues prevent strong chain interactions.

Over the past few years, many review articles have been published on guar gum and its derivatives, either used in food, nutrition, cosmetics, and in the biomedical field or in industry. However, there is no specific review reporting on guar gum-based materials for wound-healing applications to date, based on the search on the PubMed database using guar gum and wound healing as keywords that returned only 27 research articles. Taking this into consideration, the next section is divided into sub-sections based on the type of support prepared using guar gum and its derivatives.

#### 4.2.1. Films

Guar gum-based films used in wound healing can be loaded with bioactive substances, such as drugs and natural products. Recently, the incorporation of honey into the structure of these biomaterials was studied to control various parameters including swelling, degradation, and mechanical behaviour [72]. The conducted experiments demonstrated that incorporating honey into wound dressings improves their mechanical properties. In a recent study from this year, a film based on guar gum, gellan gum, and honey synthesized by the solvent casting method showed great biocompatibility, sustained proliferation, migration of the cells, and enhanced wound healing properties. The antioxidant activity of the honey-loaded and honey-free guar gum films determined by DPPH assay, showed good antioxidant abilities that increased with the quantity of honey. The MTT and scratch assays of the developed film were conducted on skin fibroblasts. Increasing the honey content caused a higher degradation rate of the biomaterial and a lower swelling capacity [72]. Another study, available as a preprint (meaning that it has not been peer reviewed by the journal), combined gelatin, bacterial nanocellulose, aldehyde-modified guar gum with decreased water solubility and honey to obtain an antibacterial film for wound healing. Similarly, the studies carried out on NIH 3T3 cells revealed biocompatibility of the support. The antimicrobial activity evaluated the inhibition activity against *Staphylococcus aureus* and *E. coli* [73].

In addition to the compounds that postulate the enhanced physicochemical properties of the biomaterial, several studies focused on developing novel formulations to integrate agents with antibacterial activity. Infections are one of the main factors influencing wound healing*, Staphylococcus aureus*, *Pseudomonas aeruginosa*, and β-hemolytic streptococci being some of the common microorganisms present in wounds [74]. Thus, the use of antibiotics and natural products with antibacterial activity could speed up the healing process. A curcumin-loaded film was synthesized using carboxymethylated guar gum grafted with gelatin, with a tensile strength of 41.64 MPa, high hemocompatibility, and biocompatibility on NIH 3T3 cells. Curcumin had a desirable release rate (93.5% in 96 h), capable of preventing bacterial development for an extended period of time [75]. Another study designed a curcumin-loaded film consisting of a guar gum/poly (3-hydroxybutyrate-co-3-hydroxyvalerate) (PHBV) blend. Biocompatibility and wound-healing properties were confirmed through MTT assay on NIH 3T3 cells, a hemolytic assay using human blood, and in vivo studies on male Swiss-albino mice carried out for 7 days. Due to considerable cell proliferation and maturation, the synthesized film improved wound healing by approximately 90%. Regarding bactericidal activity, the curcumin-loaded film was more effective on Gram-positive than Gram-negative ones. The previously demonstrated properties, along with tensile strength, swelling ratio, thermal endurance, and mechanical resistance, recommend this complex as a suitable wound dressing [76].

Other studies concentrated on the development of antibiotic-loaded films. Ceftazidime, a third-generation cephalosporin antibiotic, also used as the cross-linker, was successfully loaded into a scaffold based on aminated carboxymethyl guar gum and fish scale collagen, with a drug release of 91.3% in 96 h. MTT and CalceinAM assays evaluated the cytotoxicity in NIH 3T3 fibroblasts. The cells with 90% viability showed no morphological alterations. As a result of the antibiotic loading, the guar gum-based dressing was efficient against *Staphylococcus aureus* and *Pseudomonas aeruginosa* [77]. A carboxymethyl guar gum film loaded with Ciprofloxacin obtained by cross-linking with citric acid and tested on Wistar rats over a 5-day timeframe showed outstanding wound healing activity. The released drug was investigated at 37 °C in pH 6.8 phosphate buffer, showing a controlled release after 90 min [78]. Through microwave-assisted polycondensation with dimer acid of hydrophobically modified guar gum blended with polyvinyl alcohol, two biodegradable drug-loaded films were obtained, one loaded with Gentamicin sulfate and the other with Amoxicilin. In vivo studies carried out on Wistar albino rats showed almost full recovery of the wounds within 15 days, with the wound exudates absorbed by both films. Effective against *E. coli* and *Staphylococcus aureus*, the films also had antifungal activity on *Candida albicans* [79]. The antioxidant activity was not reported for any of these studies.

A recent patent describes the preparation of dissolvable polymeric films based on hydroxypropyl-guar, hyaluronic acid, polyethylene glycol, and polyvinylpyrrolidone capable of promoting the healing of corneal wounds. In vivo studies on a GvHD mouse model confirmed faster wound healing in mice treated with the polymer membrane, compared with those treated with lotepradnol etabonate, sodium hyaluronate, and saline [80].

#### 4.2.2. Hydrogels and Matrices

One study developed a collagen matrix cross-linked with hydrophilic polyurethane, in which guar gum was later added through semi-interpenetration. This technique conferred the polymeric matrix as having a superabsorbent behaviour, interconnected porosity, and an amorphous fibrillar structure. The authors investigated how the gelation rate, physicochemical cross-linking, swelling degree, and mechanical and thermal resistance of the semi-interpenetrating network, could be adjusted by modifying the guar gum concentration. The authors recommend these hydrogels as dressings for chronic wound healing, but no tests related to drug release, cell viability and proliferation, cell signalling, hemocompatibility, antibacterial, and antioxidant activity were performed [81]. Another interpenetrating polymeric network was obtained using carrageenan and guar gum. The ex vivo studies of the dressing revealed its adhesion to digestive tract mucosa (stomach, small intestine, large intestine). The material was tested on male mice, observing the healing of the wounds for 15 days. Histological procedures confirmed the effective recovery of the wounds [82].

An interesting study suggested a dual-layer wound dressing containing a hydrogel based on guar gum, polyvinyl alcohol, and *Lactobacillus plantarum* as the inner layer covered with a hydrocolloid based on liquid paraffin, polyisobutylene, styrene-isoprene-styrene, and sodium carboxymethylcellulose for the outer layer. Accelerated wound recovery with complete re-epithelialization was confirmed by in vivo investigations on a Pseudomonas aeruginosa-infected male Sprague-Dawley rats model [83].

Chitosan/guar gum/polyvinyl alcohol pH-responsive hydrogel blended with tetra orthosilicate was loaded with paracetamol for potential use as a wound dressing. In 140 min, the system had a drug release of 98% in PBS media, reaching a maximum swelling of 2159% in water. Tested on various bacterial strains such as *Staphylococcus aureus*,* Bacillus cereus*,* Pseudomonas aeruginosa*, and *E. coli*, the hydrogel showed excellent antibacterial activity [84]. Another self-healing and injectable hydrogel formulation based on oxidized quaternized guar gum and carboxymethyl chitosan managed to treat infected wounds of *Staphylococcus aureus* in male rats. Cytocompatibility, hemocompatibility, injectability, hemostatic, and self-healing properties are features of a promising wound dressing [85]. Guar gum/arabinoxylan-based hydrogel loaded with silver sulfadiazine exhibited an effective antifungal activity against *Pseudomonas aeruginosa* and *Staphylococcus aureus*. Having a constant drug release after 80 min with 92% of the antibiotic already released by this point, the hydrogel did not present toxicity towards MC3T3-E1 mice cells [86].

Although many studies focus on the synthesis of biomaterials that can be used as wound dressings, most of them are not designed for application on stretchable body parts. Li et al. [87] suggested the preparation of a flexible self-healing gel composed of cationic guar gum that promotes wound healing in areas prone to stretching. Recovery of the incisions made on the nape and the dorsum of the female Sprague Dawley rats confirmed the material’s capability to be an effective wound dressing while there is frequent movement of the injured area.

In 2020, novel biomaterials based on low-toxicity borax-guar gum hydrogels incorporated with curcumin-stabilized silver nanoparticles were created by Talodthaisong et al. [88]. The composite hydrogels displayed activity against *E. coli*, *Pseudomonas aeruginosa*, and *Staphylococcus aureus*, and self-healing properties on cut samples. Using the previously synthesised silver nanoparticles stabilized with curcumin, Bhubhanil et al. [89] incorporated the nanostructures into a guar gum-based hydrogel matrix. The biopolymer acted as a gelator, offering the possibility to be injectable. In vivo tests on male Wistar rats, together with in vitro investigations on dermal fibroblast cells confirmed the biocompatibility and healing properties of the hydrogel.

The same concept was followed by Ghosh Auddy et al. [90] that embedded silver nanoparticles in a guar gum alkylamine matrix. The nanobiomaterial proved faster healing and improved cosmetic appearance by combining the antibacterial properties of the nanostructures, as well as the polymer’s capacity to provide structural support. Investigating biochemical markers, such as total protein, DNA, and hydroxyproline contents of the wound tissues, the results demonstrate that the incorporation of nanoparticles into a polymeric matrix could be an excellent design for a wound dressing.

Medicinal plants were successfully incorporated into a guar gum/polyvinyl alcohol nanofibrous matrix formed through the electrospinning method. Testing the material on Wistar rats’ wounds confirmed that the tissue was completely healed and was non-toxic for gingival mesenchymal stem cells. The matrix had a tensile strength (38.78 MPa) similar to the human skin and was capable of absorbing the exudate from the wound site [91]. The electrospinning method was also used by another group of researchers to synthesize a nanofiber scaffold using carboxymethyl guar gum, reduced graphene oxide, and polyvinyl alcohol in order to obtain high-grade porosity that facilitates the transport of oxygen to cells. In vivo studies were conducted on New Zealand rabbits for 14 days, promoting the closure of the tissue excision site [92].

#### 4.2.3. Other Types of Formulations

Haynes et al. [93] patented a freeze-dried sponge synthesis technique based on xanthan and galactomannans, including guar gum, with application in wound care. To evaluate the wound healing potential of the guar gum nanoparticles synthesized in their previous work [94], Ghosh SK et al. [95] used this system in order to treat oxazolone-induced atopic dermatitis in Balb/c mice, alleviating the symptomatology. Furthermore, the findings were sustained by in vitro investigations on NIH 3T3 Scratch Assay, which displayed an effective wound recovery.

Y. Horii et al. [96] demonstrated the benefits of consuming guar gum-based dietary fibre in patients suffering from colonic epithelial cell wounds, which are frequently associated with inflammatory bowel diseases. The process is the modulated activation of an ERK1/2 and Rho kinase.

Table 2 briefly describes various formulations based on guar gum for the first time, mentioning only a few tests through which the novel biomaterials were subjected.

**Table 2:** Guar gum-based vehicles with potential use as wound dressings.

Formulation Type	Co-Products	Active Ingredient/Drug	Obtaining Method	Studies In Vitro/In Vivo	Properties	References
Film	Guar and gellan gums	Honey	Solvent casting method	DPPH assay, MTT, scratch test on skin fibroblasts	Antioxidant properties, higher honey concentration caused an increased degradation rate, a decreased swelling capacity, and a lower rate of water vapor permeability	[72]
	Aldehyde guar gum, gelatin, bacterial nanocellulose	Honey	Blending	MTT Assay, Cell attachment study on NIH 3T3 fibroblast cells	Swelling behaviour influenced by the honey concentration, antibacterial activity, improved mechanical properties	[73]
	Carboxymethyl guar gum, gelatin	Curcumin	Non-covalent interaction method	MTT Assay on NIH 3T3 fibroblast cell	Hemocompatibility, antibacterial properties	[75]
Film	Guar gum, polyhydroxyalkanoates (PHBV)	Curcumin	Solvent casting method	MTT Assay on NIH 3T3 fibroblast cell In vivo studies on Swiss-albino mice	Hemocompatibility, bactericidal activity, the PHBV content increased film’s porosity, improved mechanical properties	[76]
	Aminated carboxymethyl guar gum, collagen	Ceftazidime	Cross-linking with ceftazidime	MTT and CalceinAM assays on NIH 3T3 fibroblast	Hemocompatibility and antimicrobial activity	[77]
	Carboxymethyl guar gum	Ciprofloxacin	Cross-linking with citric acid	In vivo studies on infected Wistar rats	Hemocompatibility, increasing curing temperature decreased the swelling capacity	[78]
	Hydrophobically modified guar gum, polyvinyl alcohol	Gentamicin sulphate/Amoxicillin	Microwave-assisted poly condensation with dimer acid	In vivo studies on Wistar albino rats	Antibacterial and antifungal properties, capability of absorbing wound exudates, biodegradability	[79]
	Hydroxypropyl-guar, hyaluronic acid, PEG and polyvinylpyrrolidone	-	Film casting method	In vivo studies on GVHD mouse model (C3H mice with bone marrow and T-cells transplated from BL/6 donor)	Mucoadhesivity, the mechanical properties of the film do not impact further surgery procedures	[80]
Semi-interpenetrating hydrogel	Guar gum, collagen	-	Cross-linking with hydrophilic polyurethane	-	Superabsorbent behaviour, interconnected porosity, potential use as a wound dressing	[81]
Micro-porous interpenetrating network	Guar gum, Carrageenan	-	Microwave irradiation and film casting method	In vivo studies on mice	Hemocompatibility, degradability, honeycomb structure, good adhesion to stomach, large intestine, and small intestine	[82]
Dual-layer wound dressing	Guar gum, polyvinyl alcohol, hydrocolloid	*Lactobacillus plantarum*	Freeze-and-thaw method	In vivo studies on Male Sprague–Dawley rats	Antibacterial properties (eradicating *P. aeruginosa*), efficient in promoting complete re-epithelization	[83]
Hydrogel	Guar gum, chitosan, polyvinyl alcohol	Paracetamol	Cross-linking with tetra orthosilicate	-	Antibacterial properties, degradability, responsive to pH, potential use as a wound dressing	[84]
	Oxidized quaternized guar gum, carboxymethyl chitosan	-	Schiff base reaction	MTT Assay on L929 fibroblast cells In vivo studies on male rats with S. aureus infected wounds	Hemocompatibility, hemostatic properties, injectability, self-healing properties, antibacterial properties	[85]
	Guar gum, arabinoxylan	Silver sulfadiazine	Cross-linking with tetraethyl orthosilicate	Neutral Red assay on MC3T3-E1 cell line	Degradability, antibacterial properties	[86]
	Cationic guar gum, poly (3,4-ethylenedioxythiophene), poly(styrenesulfonate)	-	Electrostatic interaction	CCK-8 assay on NIH 3T3, MDCK and HAF cell lines In vivo studies on Sprague Dawley rats	Hemolytic activity, self-healable, conductivity, injectability, suitable for wounds located in stretchable parts of the body	[87]
Hydrogel-nanoparticle	Guar gum, curcumine-stabilisez silver nanoparticles	-	Gelation with guar gum, cross-linking with borax	Swelling degree, antimicrobial activity on *E. coli*, Pseudomonas aeruginosa, and *Staphylococcus aureus*	Improved degree of swelling, stronger inhibition against Gram-positive bacteria compared to Gram-negative bacteria	[88]
				In vivo studies on Wistar rats	Superior wound healing and antibacterial action compared to commercial gels (by >40% and 60%, respectively)	[89]
Nanobiomaterial matrix	Guar gum alkylamine, silver nanoparticles	-	In situ incorporation	In vivo studies on Wistar rats; primary skin irritation study conducted on albino rabbit	Promote wound healing by modulation of collagen deposition and regulation of keratinocytes and support re-epithelialization	[90]
Nanofibrous matrix	Guar gum, polyvinyl alcohol	*Acalypha indica*, *Aristolochia bracteolata*, *Lawsonia inermis*, and *Thespesia populne*	Electrospinning	MTT an Calcein AM assays on gingival mesenchymal stem cells, in vivo studies on Wistar rats	Exudate absorbability, tissue fully regenerated with minimal scarring	[91]
Nanofiber scaffold	Guar gum, reduced graphene oxide, polyvinyl alcohol	-		Scratch assay on 3T3-L1 fibroblast cells, In vivo studies on adult New Zealand rabbits	Oxygen transport to the cells facilitated by the porosity of the material; the moisture content of the wound site is maintained	[92]
Sponge	Xanthan, guar gum/locust bean gum	-	Freeze drying	-	Solid, bioabsorbable	[93]
Nanoparticles	Guar gum	-	Acid hydrolysis technique	Scratch assay on NIH 3T3 fibroblast cell line In vivo studies on Balb/c mice with Oxazolon-induced atopic dermatitis	Efficient in alleviating symptoms of atopic dermatitis	[94,95]
Dietary fiber	Commercial partially hydrolyzed guar gum (Sunfiber ^R^)	-	-	RhoA pull-down assays and western blot analysis on YAMC cell line, In vivo studies on C57B6 mice	Efficiency in activating ERK1/2 and Rho kinase, enhancing curing of colonic epithelial wounds	[96]

**Figure 4:**
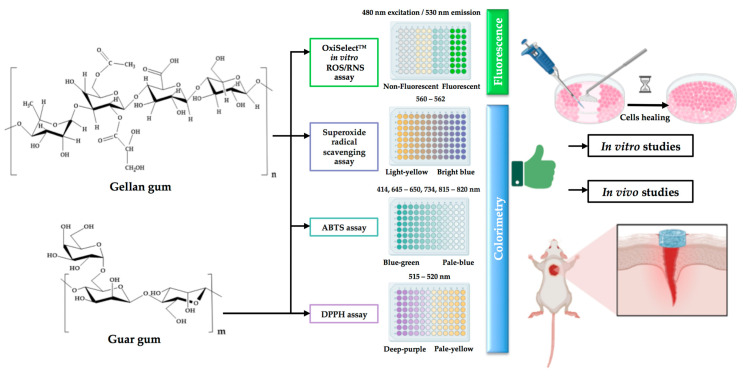
Current status on the antioxidant activity related to wound healing of gellan and guar gums: identified antioxidant activity techniques used to determine the antioxidant capacity of gellan and guar gum structures and performed studies on wound dressings based on gellan and guar gums (created with BioRender.com (accessed on 30 July 2023)).

As observed in Table 2, Figure 4, and the related description, only one support based on guar and gellan gums with incorporated honey was evaluated from the antioxidant point of view (see also Figure 3 for the detailed protocol).

The rest of the studies focused more on antibacterial activity related to wound closure, even if the healing process is modulated by the antioxidant scavenging activity. Thus, can these studies claim the critical role of ROS in wound healing and infection control at the wound site? Or can the demonstrated guar gum antioxidant activity in food packing, for example, be correlated with a complete re-epithelisation of the damaged tissue and balance of ROS and antioxidants at an optimal level? The first step was made by Kalachaveedu et al. [91], who studied guar gum medicinal plant nanofibrous mats with medicinal plants well known for their antioxidant potential. Sharma et al. [97] enlisted several medicinal plants related to being effective in the treatment of wounds in 2021. If we analyze the review paper, from the medicinal plants and their metabolites used in the treatment of different types of wounds, we could depict several, for example, curcumin, which is already incorporated into guar gum films and patented in regenerative medicine. Does this support reach the balance between ROS and antioxidants, or does another compound need to be added? Further studies must be performed in order to determine guar gum or gellan gum’s oxidative stress levels.

## 5. Conclusions

To date, wound healing and customized dressing strategies create high expectations, which are partially met by current technologies. Thus, new tools are rapidly advancing in order to achieve suitable natural-based assemblies that mimic the complex human body. It is widely acknowledged that the balance between oxidative stress and antioxidants is considered to play a fundamental role in wound dressing design. In normal conditions, low levels of ROS and oxidative stress are postulated for wound closure, but, when present in excess, lead to impaired wound healing by cell injury and inflammation, generated by oxidative stress. This review first summarised the main antioxidant assays available to be tested along with the importance of antioxidants in the wound-healing process. Then, in order to demonstrate the progressive importance of antioxidant capability, biopolymer-based gellan and guar gums were described in different tissue engineering settings. Gellan gum was found in various structures with intense wound-healing effects and quantified antioxidant potential, while guar gum was barely initiated in the field. Based on these, there is clear evidence to suspect that the antioxidant level in gum-based structures is essential in wound care and must be properly analysed in the near future.

## Figures and Tables

**Table 1:** Gellan gum-based vehicles with potential use as wound dressings.

Formulation Type	Co-Products	Active Ingredient/Drug	Obtaining Method	Studies In Vitro/In Vivo	Properties	References
Spongy-like hydrogel	Hyaluronic acid/Ca^2+^	Human dermal/epidermal cell fractions	Ionic cross-linking	In vivo mice wound healing	Accelerated rate of wound closure, re-epithelization, and tissue neovascularization	[48]
	Calcium chloride (CaCl_2_)	Eumelanin	Ionic cross-linking	Oxi-Select in vitro ROS and reactive nitrogen species (RNS) assay, MTS assay, in vivo host reaction in mice	Sustained release of eumelanin and antioxidant property	[49]
Hydrogel	Chitosan/PEG	Apigenin	Covalent cross-linking	In vivo rat diabetic wound healing, collagen content, protein level and granuloma weight measurement, biochemical parameters, SOD, GSH, and catalase for antioxidant levels	Biocompatibility, biodegradability, entrapment and sustained release of apigenin, moist nature, enhanced rate of wound contraction and reduced healing time, antioxidant effectiveness	[50]
	Carboxymethyl chitosan	Bovine serum albumin (BSA)/tetracycline hydrochloride/silver sulfadiazine	Schiff-base cross-linking	In vitro antibacterial activity	Sustained release of drugs and strong antibacterial activity	[51]
	Fucoidan/alginate gum/carboxymethyl cellulose	Chlorin-e6	Chemical cross-linking	In vitro cytotoxicity (MTT and LDH) and cell proliferation assay (BrdU), determination of ROS production (DPPH assay), evaluation of singlet oxygen (DPBF assay), in vitro wound healing (scratch assay), in vivo mice wound healing	Excellent biocompatibility, antioxidant property, and accelerated rate of wound closure	[52]
	4 arm-PEG-Vinylsulfone/polydopamine	Ciprofloxacin	Michael addition	In vitro cytotoxicity (MTS) and live/dead assay, in vitro antimicrobial activity	Antimicrobial activity	[53]
	Sodium alginate/CaCl_2_	Nisin	Ionic cross-linking	In vitro cytotoxicity (alamarBlue) and live/dead staining assay, in vitro antimicrobial activity	Cytocompatibility and antimicrobial activity	[54]
	Methacrylate anhydride	-	Thiol-ene photoclick chemistry	In vitro live/dead assay, immunocytochemistry analysis (α-SMA and focal adhesion staining)	Suitable for controlling scar tissue formation	[55]
	Propylene glycol/CaCl_2_	Ofloxacin/lavender oil/tea tree oil	Solvent casting ionotropic gelation	In vitro antibacterial activity, DPPH radical scavenging assay, in vivo rat wound healing	Potent antibacterial activity, accelerated rate of wound closure, and re-epithelization	[56]
	Polyacrylamide/Mg^2+^	-	Free radical polymerization	In vitro cytotoxicity assay (MTT), Western blot assays, in vivo rat burn wound model	Good biocompatibility and accelerated rate of wound healing	[57]
	Sodium alginate/Ca^2+/^polydopamine	Doxorubicin	3D printing	In vitro cytotoxicity (MTT) and live/dead staining assay, in vivo mice wound healing	Good cytocompatibility and promoted wound healing following surgery	[58]
	Ca^2+^	Human-derived bone mesenchymal stem cell	Ionic crosslinking and guest-host interaction	In vitro cytotoxicity (MTT) and live/dead staining assay, in vitro wound healing (scratch assay), ELISA assay (IGF-1, HGF, TGF-β1, and VEGF)	Enhanced cell growth and promising candidate for regenerative medicine	[59]
	PEG/polydopamine	Colistin	Electrostatic interaction	In vitro antioxidant activity (DPPH and ABTS•+ radical scavenging assay), in vitro cytotoxicity (MTS), and live/dead staining assay, in vitro antibacterial activity	Excellent cytocompatibility and antioxidant activity	[60]
	Sericin/halloysite/polydopamine	-	Co-blending	In vitro antibacterial activity, cytotoxicity (CCK-8), and live/dead staining assay, in vivo bacteria-infected wound healing in rat	Rapid hemostasis and promoted infected wound repair	[61]
Nanohydrogel	Cholesterol	Baicalin	Self-assembly	In vivo mice anti-inflammatory and skin repair tests	Effective delivery system for the prevention and treatment of inflamed and/or damaged skin	[62]
Film	CaCl_2_/titanium dioxide (TiO_2_)	-	Evaporative casting	In vitro antibacterial activity and wound healing, in vivo rat wound healing	Enhanced antibacterial properties, cell viability and cell proliferation, accelerated rate of wound healing	[63]
	TiO_2_	-	Evaporative casting	In vitro antibacterial activity and cytotoxicity assay (MTT), in vivo rat wound healing	Antibacterial activity, good biocompatibility, and accelerated rate of wound closure	[64]
	Chitosan/N,N,N-trimethyl chitosan/iota-carrageenan	Tanfloc	Solvent evaporation	Bactericidal and anti-adhesive assays, in vitro cytotoxicity assay (alamarBlue)	Antimicrobial and cytocompatibility properties	[65]
	Zinc oxide/carbon nanotubes	-	Solvent casting	In vitro antibacterial activity, in vivo rat wound healing	Good antibacterial activity, promoted complete wound healing	[66]
Membrane	Bacterial cellulose/xyloglucan/lysozyme/mesenchymal stem cell	-	Dry-cast process	In vitro cytotoxicity assay (MTT), in vivo rat burn wound healing, immunocytochemistry assays (BrdU and DAPI)	Reduced acute/chronic inflammatory infiltrates, accelerated rate of wound healing, and re-epithelization	[67]
Full-IPN hydrogel	Type-1 collagen/magnesium chloride (MgCl_2_)/adipose-derived mesenchymal stem cell	-	Ionic cross-linking	In vitro cytotoxicity assay (MTS) and wound healing (scratch assay), in vivo murine burn wound model, ELISA assay (human TSG-6)	Enhanced early wound closure, reducing inflammation, and promoting complete skin regeneration	[68]
Fiber	ε-poly-L-lysine/glucose/soybean peptide/fish collagen peptide	-	Wet spinning	In vitro antibacterial activity and cytotoxicity (MTT), In vivo rat wound healing	Good cytocompatibility and antibacterial activity, effectively promoted wound healing	[69]

## Data Availability

All data is available upon request.

## Abbreviations

AAPH: 2,2′ -azobis-2-amidino-propane; ABTS: 2,2′-azinobis-(3- ethylbenzothiazoline-6-sulfonic acid); BSA: Bovine serum albumin; BrdU: 5-bromodeoxyuridine; CaCl_2_: Calcium chloride; CAT: catalase assay; CUPRAC: cupric reducing antioxidant power; CCK-8: Cell Counting Kit-8; DAPI: 4′,6-diamidino-2-phenylindole; DPPH: 2,2-di(4-tert-octylphenyl)-1-picrylhydrazyl; DPBF: 1,3-Diphenylisobenzofuran; DCFH: 2′,7′-dichlorodihydrofluorescein diacetate; ECM: extracellular matrix; EC_50_: the efficient concentration of the antioxidant necessary to reduce the initial DPPH concentration by 50%; EDTA: ethylenediamine tetraacetic acid; ELISA: enzyme-linked immunosorbent assay; Fe (III): ferric ions; Fe (II): ferrous complex; FOX: ferrous oxidation xylenol; FTC: ferric thiocyanate; FRAP: ferric reducing antioxidant power; GAE: gallic acid equivalents; GvHD: Graft vs Host Disease; GSH: glutathione; GSH-Px: glutathione peroxidase; HGF: hepatocyte growth factor; HORAC: hydroxyl radical antioxidant capacity; IGF-1: insulin-like growth factor; KOH: potassium hydroxide; LDH: lactate dehydrogenase; MDA: malondialdehyde; MTT: 3-(4,5-dimethylthiazol-2-yl)-2,5-diphenyl-2H-tetrazolium bromide; MTS: 3-(4,5-dimethylthiazol-2-yl)-5-(3-carboxymethoxyphenyl)-2-(4-sulfophenyl)-2H-tetrazolium bromide; NaOH: sodium hydroxide; NBT: nitroblue tetrazolium; NED: N-(1-naphthyl) ethylenediamine; ORAC: oxygen radical absorption capacity; O_2_: oxygen; PBS: phosphate buffer saline; PEG: poly ethylene glycol; RNS: reactive nitrogen species; ROS: reactive oxygen species; SMA: smooth muscle actin; SNP: sodium nitroprusside; SOD: superoxide dismutase; TAC: total antioxidant activity; TBA: thiobarbituric acid; TBARS: thiobarbituric acid reactive substances; TEC_50_: the necessary time to reach the equilibrium state with EC_50_; TGF-β1: transforming growth factor-beta; TiO_2_: titanium dioxide; TPC: total phenolic content; TRAP: total peroxyl radical trapping antioxidant parameter; UV-VIS: ultraviolet visible; VEGF: vascular endothelial growth factor; XO: xylenol orange.

## References

[1] Farahani M., Shafiee A. (2021). Wound Healing: From Passive to Smart Dressings. Adv. Healthc. Mater..

[2] Gardikiotis I., Cojocaru F.-D., Mihai C.-T., Balan V., Dodi G. (2022). Borrowing the Features of Biopolymers for Emerging Wound Healing Dressings: A Review. Int. J. Mol. Sci..

[3] Nejaddehbashi F., Rafiee Z., Orazizadeh M., Bayati V., Hemmati A., Hashemitabar M., Makvandi P. (2023). Antibacterial and Antioxidant Double-Layered Nanofibrous Mat Promotes Wound Healing in Diabetic Rats. Sci. Rep..

[4] Agyare C., Bekoe E.O., Boakye Y.D., Dapaah S.O., Appiah T., Bekoe S.O., Alexandrescu V.A. (2016). Medicinal Plants and Natural Products with Demonstrated Wound Healing Properties. Wound Healing—New insights into Ancient Challenges.

[5] Brainina K., Stozhko N., Vidrevich M. (2019). Antioxidants: Terminology, Methods, and Future Considerations. Antioxidants.

[6] Hajhashemi V., Vaseghi G., Pourfarzam M., Abdollahi A. (2010). Are Antioxidants Helpful for Disease Prevention?. Res. Pharm. Sci..

[7] Bardia A., Tleyjeh I.M., Cerhan J.R., Sood A.K., Limburg P.J., Erwin P.J., Montori V.M. (2008). Efficacy of Antioxidant Supplementation in Reducing Primary Cancer Incidence and Mortality: Systematic Review and Meta-Analysis. Mayo Clin. Proc..

[8] Sharifi-Rad M., Anil Kumar N.V., Zucca P., Varoni E.M., Dini L., Panzarini E., Rajkovic J., Tsouh Fokou P.V., Azzini E., Peluso I. (2020). Lifestyle, Oxidative Stress, and Antioxidants: Back and Forth in the Pathophysiology of Chronic Diseases. Front. Physiol..

[9] Rudrapal M., Khairnar S.J., Khan J., Dukhyil A.B., Ansari M.A., Alomary M.N., Alshabrmi F.M., Palai S., Deb P.K., Devi R. (2022). Dietary Polyphenols and Their Role in Oxidative Stress-Induced Human Diseases: Insights into Protective Effects, Antioxidant Potentials and Mechanism(s) of Action. Front. Pharmacol..

[10] Tabriz A.G., Douroumis D. (2022). Recent Advances in 3D Printing for Wound Healing: A Systematic Review. J. Drug Deliv. Sci. Technol..

[11] Hajialyani M., Tewari D., Sobarzo-Sánchez E., Nabavi S.M., Farzaei M.H., Abdollahi M. (2018). Natural Product-Based Nanomedicines for Wound Healing Purposes: Therapeutic Targets and Drug Delivery Systems. IJN.

[12] LaVan F.B., Hunt T.K. (1990). Oxygen and Wound Healing. Clin. Plast. Surg..

[13] Hopf H.W. (2003). Development of Subcutaneous Wound Oxygen Measurement in Humans: Contributions of Thomas, K. Hunt, MD. Wound Repair Regen.

[14] Gordillo G.M., Sen C.K. (2003). Revisiting the Essential Role of Oxygen in Wound Healing. Am. J. Surg..

[15] Dunnill C., Patton T., Brennan J., Barrett J., Dryden M., Cooke J., Leaper D., Georgopoulos N.T. (2017). Reactive Oxygen Species (ROS) and Wound Healing: The Functional Role of ROS and Emerging ROS-Modulating Technologies for Augmentation of the Healing Process: Reactive Oxygen Species and Wound Healing. Int. Wound J..

[16] Shen H.-M., Pervaiz S., Dong Z., Yin X.-M. (2009). Reactive Oxygen Species in Cell Fate Decisions. Essentials of Apoptosis.

[17] Poljsak B., Šuput D., Milisav I. (2013). Achieving the Balance between ROS and Antioxidants: When to Use the Synthetic Antioxidants. Oxidative Med. Cell. Longev..

[18] Rodriguez P.G., Felix F.N., Woodley D.T., Shim E.K. (2008). The Role of Oxygen in Wound Healing: A Review of the Literature. Dermatol. Surg..

[19] Munteanu I.G., Apetrei C. (2021). Analytical Methods Used in Determining Antioxidant Activity: A Review. Int. J. Mol. Sci..

[20] Christodoulou M.C., Orellana Palacios J.C., Hesami G., Jafarzadeh S., Lorenzo J.M., Domínguez R., Moreno A., Hadidi M. (2022). Spectrophotometric Methods for Measurement of Antioxidant Activity in Food and Pharmaceuticals. Antioxidants.

[21] Moharram H., Youssef M. (2014). Methods for Determining the Antioxidant Activity: A Review. Alex. J. Fd. Sci. Technol..

[22] Carocho M., Ferreira I.C.F.R. (2013). A Review on Antioxidants, Prooxidants and Related Controversy: Natural and Synthetic Compounds, Screening and Analysis Methodologies and Future Perspectives. Food Chem. Toxicol..

[23] Si Trung T., Bao H.N.D. (2015). Physicochemical Properties and Antioxidant Activity of Chitin and Chitosan Prepared from Pacific White Shrimp Waste. Int. J. Carbohydr. Chem..

[24] Everette J.D., Bryant Q.M., Green A.M., Abbey Y.A., Wangila G.W., Walker R.B. (2010). Thorough Study of Reactivity of Various Compound Classes toward the Folin−Ciocalteu Reagent. J. Agric. Food Chem..

[25] Amoli P., Hadidi M., Hasiri Z., Rouhafza A., Jelyani A., Hadian Z., Khaneghah A., Lorenzo J. (2021). Incorporation of Low Molecular Weight Chitosan in a Low-Fat Beef Burger: Assessment of Technological Quality and Oxidative Stability. Foods.

[26] Gulcin İ. (2020). Antioxidants and Antioxidant Methods: An Updated Overview. Arch Toxicol..

[27] Aguilar Diaz De Leon J., Borges C.R. (2020). Evaluation of Oxidative Stress in Biological Samples Using the Thiobarbituric Acid Reactive Substances Assay. JoVE.

[28] del Carmen Pinto M., Tejeda A., Duque A.L., Macías P. (2007). Determination of Lipoxygenase Activity in Plant Extracts Using a Modified Ferrous Oxidation−Xylenol Orange Assay. J. Agric. Food Chem..

[29] Sharma S., Vig A.P. (2013). Evaluation of in Vitro Antioxidant Properties of Methanol and Aqueous Extracts of *Parkinsonia aculeata*, L. Leaves. Sci. World J..

[30] Moon J.-K., Shibamoto T. (2009). Antioxidant Assays for Plant and Food Components. J. Agric. Food Chem..

[31] Hazra B., Biswas S., Mandal N. (2008). Antioxidant and Free Radical Scavenging Activity of Spondias Pinnata. BMC Complement. Altern. Med..

[32] Santos-Sánchez N.F., Salas-Coronado R., Villanueva-Cañongo C., Hernández-Carlos B., Santos-Sánchez N.F., Salas-Coronado R., Villanueva-Cañongo C., Hernández-Carlos B. (2019). Antioxidant Compounds and Their Antioxidant Mechanism. Antioxidants.

[33] Bibi Sadeer N., Montesano D., Albrizio S., Zengin G., Mahomoodally M.F. (2020). The Versatility of Antioxidant Assays in Food Science and Safety—Chemistry, Applications, Strengths, and Limitations. Antioxidants.

[34] Apak R., Capanoglu E., Shahidi F. (2017). Measurement of Antioxidant Activity and Capacity: Recent Trends and Applications.

[35] Li Y., Schellhorn H.E. (2007). Rapid Kinetic Microassay for Catalase Activity. J. Biomol. Tech..

[36] Ursini F., Maiorino M. (2013). Glutathione Peroxidases. Encyclopedia of Biological Chemistry.

[37] Prasad N., Ramteke P., Dholia N., Yadav U.C.S. (2018). Therapeutic Interventions to Block Oxidative Stress-Associated Pathologies. Immunity and Inflammation in Health and Disease.

[38] Rahman I., Kode A., Biswas S.K. (2006). Assay for Quantitative Determination of Glutathione and Glutathione Disulfide Levels Using Enzymatic Recycling Method. Nat. Protoc..

[39] Rana V., Rai P., Tiwary A.K., Singh R.S., Kennedy J.F., Knill C.J. (2011). Modified Gums: Approaches and Applications in Drug Delivery. Carbohydr. Polym..

[40] Nagaraja K., Rao K.M., Krishna Rao K.S.V., Riazunnisa K., Suresh Reddy K.V.N., Kim J.-C., Alle M., Husen A. (2021). Polysaccharides of Natural Gums-Based Biomedical Devices for Drug Delivery Application. Smart Nanomaterials in Biomedical Applications.

[41] Mano J.F., Silva G.A., Azevedo H.S., Malafaya P.B., Sousa R.A., Silva S.S., Boesel L.F., Oliveira J.M., Santos T.C., Marques A.P. (2007). Natural Origin Biodegradable Systems in Tissue Engineering and Regenerative Medicine: Present Status and Some Moving Trends. J. R Soc. Interface.

[42] Mohammadinejad R., Kumar A., Ranjbar-Mohammadi M., Ashrafizadeh M., Han S.S., Khang G., Roveimiab Z. (2020). Recent Advances in Natural Gum-Based Biomaterials for Tissue Engineering and Regenerative Medicine: A Review. Polymers.

[43] Munir H., Bilal M., Khan M.I., Iqbal H.M.N., Inamuddin, Ahamed M.I., Boddula R., Altalhi T. (2021). Gums-Based Bionanostructures for Medical Applications. Polysaccharides.

[44] Giavasis I., Harvey L.M., McNeil B. (2000). Gellan Gum. Crit. Rev. Biotechnol..

[45] Zia K.M., Tabasum S., Khan M.F., Akram N., Akhter N., Noreen A., Zuber M. (2018). Recent Trends on Gellan Gum Blends with Natural and Synthetic Polymers: A Review. Int. J. Biol. Macromol..

[46] Feketshane Z., Alven S., Aderibigbe B.A. (2022). Gellan Gum in Wound Dressing Scaffolds. Polymers.

[47] Gomes D., Batista-Silva J.P., Sousa A., Passarinha L.A. (2023). Progress and Opportunities in Gellan Gum-Based Materials: A Review of Preparation, Characterization and Emerging Applications. Carbohydr. Polym..

[48] Cerqueira M.T., Da Silva L.P., Santos T.C., Pirraco R.P., Correlo V.M., Marques A.P., Reis R.L. (2014). Human Skin Cell Fractions Fail to Self-Organize Within a Gellan Gum/Hyaluronic Acid Matrix but Positively Influence Early Wound Healing. Tissue Eng. Part A.

[49] Da Silva L.P., Oliveira S., Pirraco R.P., Santos T.C., Reis R.L., Marques A.P., Correlo V.M. (2017). Eumelanin-Releasing Spongy-like Hydrogels for Skin Re-Epithelialization Purposes. Biomed. Mater..

[50] Shukla R., Kashaw S.K., Jain A.P., Lodhi S. (2016). Fabrication of Apigenin Loaded Gellan Gum–Chitosan Hydrogels (GGCH-HGs) for Effective Diabetic Wound Healing. Int. J. Biol. Macromol..

[51] Zhang X., Pan Y., Li S., Xing L., Du S., Yuan G., Li J., Zhou T., Xiong D., Tan H. (2020). Doubly Crosslinked Biodegradable Hydrogels Based on Gellan Gum and Chitosan for Drug Delivery and Wound Dressing. Int. J. Biol. Macromol..

[52] Shanmugapriya K., Kim H., Kang H.W. (2020). Fucoidan-Loaded Hydrogels Facilitates Wound Healing Using Photodynamic Therapy by in Vitro and in Vivo Evaluation. Carbohydr. Polym..

[53] Fiorica C., Palumbo F.S., Pitarresi G., Biscari G., Martorana A., Calà C., Maida C.M., Giammona G. (2021). Ciprofloxacin Releasing Gellan Gum/Polydopamine Based Hydrogels with near Infrared Activated Photothermal Properties. Int. J. Pharm..

[54] Reczyńska-Kolman K., Hartman K., Kwiecień K., Brzychczy-Włoch M., Pamuła E. (2021). Composites Based on Gellan Gum, Alginate and Nisin-Enriched Lipid Nanoparticles for the Treatment of Infected Wounds. Int. J. Mol. Sci..

[55] Li Z., Bratlie K.M. (2021). Fibroblasts Treated with Macrophage Conditioned Medium Results in Phenotypic Shifts and Changes in Collagen Organization. Mater. Sci. Eng. C.

[56] Mahmood H., Khan I.U., Asif M., Khan R.U., Asghar S., Khalid I., Khalid S.H., Irfan M., Rehman F., Shahzad Y. (2021). In Vitro and in Vivo Evaluation of Gellan Gum Hydrogel Films: Assessing the Co Impact of Therapeutic Oils and Ofloxacin on Wound Healing. Int. J. Biol. Macromol..

[57] Li W., Jian X., Zou Y., Wu L., Huang H., Li H., Hu D., Yu B. (2021). The Fabrication of a Gellan Gum-Based Hydrogel Loaded With Magnesium Ions for the Synergistic Promotion of Skin Wound Healing. Front. Bioeng. Biotechnol..

[58] Xu L., Chen Y., Zhang P., Tang J., Xue Y., Luo H., Dai R., Jin J., Liu J. (2022). 3D Printed Heterogeneous Hybrid Hydrogel Scaffolds for Sequential Tumor Photothermal-Chemotherapy and Wound Healing. Biomater. Sci..

[59] Choi J.H., In Kim S., Seo J.S., Tumursukh N.-E., Kim S.E., Choe S.H., Kim S.J., Park S., Song J.E., Khang G. (2022). Fast Stress Relaxing Gellan Gum That Enhances the Microenvironment and Secreting Function of Bone Mesenchymal Stem Cells. Int. J. Biol. Macromol..

[60] Biscari G., Pitarresi G., Fiorica C., Schillaci D., Catania V., Palumbo F.S., Giammona G. (2022). Near-Infrared Light-Responsive and Antibacterial Injectable Hydrogels with Antioxidant Activity Based on a Dopamine-Functionalized Gellan Gum for Wound Healing. Int. J. Pharm..

[61] Yuan L., Jiang X., Jiang M., Guo Y., Liu Y., Ming P., Li S., Zhou P., Cai R., Yu K. (2023). Biocompatible Gellan Gum/Sericin Hydrogels Containing Halloysite@polydopamine Nanotubes with Hemostasis and Photothermal Antibacterial Properties for Promoting Infectious Wound Repair. Mater. Des..

[62] Manconi M., Manca M.L., Caddeo C., Valenti D., Cencetti C., Diez-Sales O., Nacher A., Mir-Palomo S., Terencio M.C., Demurtas D. (2018). Nanodesign of New Self-Assembling Core-Shell Gellan-Transfersomes Loading Baicalin and In Vivo Evaluation of Repair Response in Skin. Nanomed. Nanotechnol. Biol. Med..

[63] Ismail N.A., Amin K.A.M., Majid F.A.A., Razali M.H. (2019). Gellan Gum Incorporating Titanium Dioxide Nanoparticles Biofilm as Wound Dressing: Physicochemical, Mechanical, Antibacterial Properties and Wound Healing Studies. Mater. Sci. Eng. C.

[64] Razali M.H., Ismail N.A., Mat Amin K.A. (2020). Titanium Dioxide Nanotubes Incorporated Gellan Gum Bio-Nanocomposite Film for Wound Healing: Effect of TiO_2_ Nanotubes Concentration. Int. J. Biol. Macromol..

[65] Rufato K.B., Souza P.R., De Oliveira A.C., Berton S.B.R., Sabino R.M., Muniz E.C., Popat K.C., Radovanovic E., Kipper M.J., Martins A.F. (2021). Antimicrobial and Cytocompatible Chitosan, N,N,N-Trimethyl Chitosan, and Tanfloc-Based Polyelectrolyte Multilayers on Gellan Gum Films. Int. J. Biol. Macromol..

[66] Liu J., Ismail N.A., Yusoff M., Razali M.H. (2022). Physicochemical Properties and Antibacterial Activity of Gellan Gum Incorporating Zinc Oxide/Carbon Nanotubes Bionanocomposite Film for Wound Healing. Bioinorg. Chem. Appl..

[67] Costa De Oliveira Souza C.M., De Souza C.F., Mogharbel B.F., Irioda A.C., Cavichiolo Franco C.R., Sierakowski M.R., Athayde Teixeira De Carvalho K. (2021). Nanostructured Cellulose–Gellan–Xyloglucan–Lysozyme Dressing Seeded with Mesenchymal Stem Cells for Deep Second-Degree Burn Treatment. IJN.

[68] Ng J.Y., Zhu X., Mukherjee D., Zhang C., Hong S., Kumar Y., Gokhale R., Ee P.L.R. (2021). Pristine Gellan Gum–Collagen Interpenetrating Network Hydrogels as Mechanically Enhanced Anti-Inflammatory Biologic Wound Dressings for Burn Wound Therapy. ACS Appl. Bio Mater..

[69] Zhang Y., Wu J., Yu K., Hu J., Zhan X. (2022). Preparation and Characterization of Bifunctional Edible Gellan-Polylysine Fiber. Int. J. Biol. Macromol..

[70] Mudgil D., Barak S., Khatkar B.S. (2014). Guar Gum: Processing, Properties and Food Applications—A Review. J. Food Sci. Technol..

[71] Prabaharan M. (2011). Prospective of Guar Gum and Its Derivatives as Controlled Drug Delivery Systems. Int. J. Biol. Macromol..

[72] Bal-Öztürk A., Torkay G., İdil N., Özkahraman B., Özbaş Z. (2023). Gellan Gum/Guar Gum Films Incorporated with Honey as Potential Wound Dressings. Polym. Bull..

[73] Nezhadmokhtari P., Asadi N., Ghorbani M., Bakhshayesh A.R.D., Milani M., Akbarzadeh A. (2021). Development of a Novel Film Based on Bacterial Nanocellulose Reinforced Gelatin/Guar Gum Containing Honey for Wound Healing Applications. Res. Sq..

[74] Guo S., DiPietro L.A. (2010). Factors Affecting Wound Healing. J. Dent. Res..

[75] Manna P.J., Mitra T., Pramanik N., Kavitha V., Gnanamani A., Kundu P.P. (2015). Potential Use of Curcumin Loaded Carboxymethylated Guar Gum Grafted Gelatin Film for Biomedical Applications. Int. J. Biol. Macromol..

[76] Pramanik N., Mitra T., Khamrai M., Bhattacharyya A., Mukhopadhyay P., Gnanamani A., Basu R.K., Kundu P.P. (2015). Characterization and Evaluation of Curcumin Loaded Guar Gum/Polyhydroxyalkanoates Blend Films for Wound Healing Applications. RSC Adv..

[77] Jana P., Mitra T., Selvaraj T.K.R., Gnanamani A., Kundu P.P. (2016). Preparation of Guar Gum Scaffold Film Grafted with Ethylenediamine and Fish Scale Collagen, Cross-Linked with Ceftazidime for Wound Healing Application. Carbohydr. Polym..

[78] Orsu P., Matta S. (2020). Fabrication and Characterization of Carboxymethyl Guar Gum Nanocomposite for Application of Wound Healing. Int. J. Biol. Macromol..

[79] Bajpai A., Raj V. (2021). Hydrophobically Modified Guar Gum Films for Wound Dressing. Polym. Bull..

[80] Ketelson H.A., Rangarajan R. (2023). Dissolvable Medical Device for Promoting Healing of Wounds. U.S. Patent.

[81] Lopéz-Martínez E.E., Claudio-Rizo J.A., Caldera-Villalobos M., Becerra-Rodríguez J.J., Cabrera-Munguía D.A., Cano-Salazar L.F., Betancourt-Galindo R. (2022). Hydrogels for Biomedicine Based on Semi-Interpenetrating Polymeric Networks of Collagen/Guar Gum: Synthesis and Physicochemical Characterization. Macromol. Res..

[82] Swain S., Bal T. (2019). Microwave Irradiated Carrageenan-Guar Gum Micro-Porous IPN: A Novel Material for Isotropic Tissue Scaffolding. Int. J. Polym. Mater. Polym. Biomater..

[83] Kim J.S., Kim J., Lee S.M., Woo M.R., Kim D.W., Kim J.O., Choi H.-G., Jin S.G. (2022). Development of Guar Gum-Based Dual-Layer Wound Dressing Containing Lactobacillus Plantarum: Rapid Recovery and Mechanically Flexibility. Int. J. Biol. Macromol..

[84] Khan M.U.A., Iqbal I., Ansari M.N.M., Razak S.I.A., Raza M.A., Sajjad A., Jabeen F., Riduan Mohamad M., Jusoh N. (2021). Development of Antibacterial, Degradable and PH-Responsive Chitosan/Guar Gum/Polyvinyl Alcohol Blended Hydrogels for Wound Dressing. Molecules.

[85] Yu X., Cheng C., Peng X., Zhang K., Yu X. (2022). A Self-Healing and Injectable Oxidized Quaternized Guar Gum/Carboxymethyl Chitosan Hydrogel with Efficient Hemostatic and Antibacterial Properties for Wound Dressing. Colloids Surf. B Biointerfaces.

[86] Khan M.U.A., Raza M.A., Razak S.I.A., Abdul Kadir M.R., Haider A., Shah S.A., Mohd Yusof A.H., Haider S., Shakir I., Aftab S. (2020). Novel Functional Antimicrobial and Biocompatible Arabinoxylan/Guar Gum Hydrogel for Skin Wound Dressing Applications. J. Tissue Eng. Regen. Med..

[87] Li S., Wang L., Zheng W., Yang G., Jiang X. (2020). Rapid Fabrication of Self-Healing, Conductive, and Injectable Gel as Dressings for Healing Wounds in Stretchable Parts of the Body. Adv. Funct. Mater..

[88] Talodthaisong C., Boonta W., Thammawithan S., Patramanon R., Kamonsutthipaijit N., Hutchison J.A., Kulchat S. (2020). Composite Guar Gum-Silver Nanoparticle Hydrogels as Self-Healing, Injectable, and Antibacterial Biomaterials. Mater. Today Commun..

[89] Bhubhanil S., Talodthaisong C., Khongkow M., Namdee K., Wongchitrat P., Yingmema W., Hutchison J.A., Lapmanee S., Kulchat S. (2021). Enhanced Wound Healing Properties of Guar Gum/Curcumin-Stabilized Silver Nanoparticle Hydrogels. Sci. Rep..

[90] Ghosh Auddy R., Abdullah M.F., Das S., Roy P., Datta S., Mukherjee A. (2013). New Guar Biopolymer Silver Nanocomposites for Wound Healing Applications. BioMed Res. Int..

[91] Kalachaveedu M., Jenifer P., Pandian R., Arumugam G. (2020). Fabrication and Characterization of Herbal Drug Enriched Guar Galactomannan Based Nanofibrous Mats Seeded with GMSC’s for Wound Healing Applications. Int. J. Biol. Macromol..

[92] Koyyada A., Orsu P. (2021). Nanofibrous Scaffolds of Carboxymethyl Guargum Potentiated with Reduced Graphene Oxide for in Vitro and in Vivo Wound Healing Applications. Int. J. Pharm..

[93] Haynes C.A., Lorimer E. (2001). Solid Polysaccharide Materials for Use as Wound Dressings. U.S. Patent.

[94] Ghosh S.K., Abdullah F., Mukherjee A. (2015). Fabrication and Fluorescent Labeling of Guar Gum Nanoparticles in a Surfactant Free Aqueous Environment. Mater. Sci. Eng. C.

[95] Ghosh N., Mitra S., Banerjee E.R. (2018). Therapeutic Effects of Topically-Administered Guar Gum Nanoparticles in Oxazolone-Induced Atopic Dermatitis in Mice. Biomed. Res. Ther..

[96] Horii Y., Uchiyama K., Toyokawa Y., Hotta Y., Tanaka M., Yasukawa Z., Tokunaga M., Okubo T., Mizushima K., Higashimura Y. (2016). Partially Hydrolyzed Guar Gum Enhances Colonic Epithelial Wound Healing via Activation of RhoA and ERK1/2. Food Funct..

[97] Sharma A., Khanna S., Kaur G., Singh I. (2021). Medicinal Plants and Their Components for Wound Healing Applications. Futur J. Pharm. Sci..

